# Identification of Sphingomyelinase on the Surface of *Chlamydia pneumoniae*: Possible Role in the Entry into Its Host Cells

**DOI:** 10.1155/2014/412827

**Published:** 2014-03-13

**Authors:** Tuula A. Peñate Medina, Juha T. Korhonen, Riitta Lahesmaa, Mirja Puolakkainen, Oula Peñate Medina, Paavo K. J. Kinnunen

**Affiliations:** ^1^Helsinki Biophysics & Biomembrane Group, Medical Biochemistry, Institute of Biomedicine, University of Helsinki, 00014 Helsinki, Finland; ^2^Molecular Imaging North Competence Center (MOIN CC), Christian-Albrechts Universität zu Kiel, AmBotanischen Garten 14, 24118 Kiel, Germany; ^3^Turku Centre for Biotechnology, 20520 Turku, Finland; ^4^HUSLAB, Department of Virology, Haartman Institute, University of Helsinki, 00014 Helsinki, Finland; ^5^Helsinki Biophysics & Biomembrane Group, Department of Biomedical Engineering and Computational Science, P.O. Box 12200, Rakentajanaukio 3, 00076 Aalto, Finland

## Abstract

We have recently suggested a novel mechanism, autoendocytosis, for the entry of certain microbes into their hosts, with a key role played by the sphingomyelinase-catalyzed topical conversion of sphingomyelin to ceramide, the differences in the biophysical properties of these two lipids providing the driving force. The only requirement for such microbes to utilize this mechanism is that they should have a catalytically active SMase on their outer surface while the target cells should expose sphingomyelin in the external leaflet of their plasma membrane. In pursuit of possible microbial candidates, which could utilize this putative mechanism, we conducted a sequence similarity search for SMase. Because of the intriguing cellular and biochemical characteristics of the poorly understood entry of *Chlamydia* into its host cells these microbes were of particular interest. SMase activity was measured *in vitro* from isolated *C. pneumoniae* elementary bodies (EB) and in the lysate from *E. coli* cells transfected with a plasmid expressing CPn0300 protein having sequence similarity to SMase. Finally, pretreatment of host cells with exogenous SMase resulting in loss plasma membrane sphingomyelin attenuated attachment of EB.

## 1. Introduction

Sphingomyelinase (SMase, sphingomyelin phosphodiesterase, E.C.3.1.4.12) is a hydrolytic enzyme, which cleaves the phosphocholine moiety from sphingomyelin, yielding ceramide. Interestingly, there is a wide range of bacteria, viruses, and parasites, which need ceramide in one way or another to enter nonphagocytic cells and to cause infection, for example,* Neisseria gonorrhoeae* [1],* Pseudomonas aeruginosa* [2],* Staphylococcus aureus *[3], Semliki Forest virus [4], Sindbis virus [5], and Rhinovirus [6]. Likewise,* Plasmodium falciparum* needs SMase for the infection of erythrocytes [7]. The molecular level mechanisms have remained elusive.* Pseudomonas* and* Neisseria* activate the SMase of the host cell but, for example, the most virulent Mycobacteria [8],* Clostridium perfringens* [9],* Bacillus cereus* [10],* Listeria monocytogenes* [11],* Helicobacter pylori* [12], and* Leptospira interrogans* [13], have endogenous SMase or sphingomyelinase-like protein.

We have previously demonstrated following a topical microinjection of SMase a vectorial formation of ceramide enriched vesicles in sphingomyelin containing giant liposome model membranes [14]. More specifically, a release of “endocytotic” vesicles into the giant liposome or vesicle budding from its outer surface was seen following the action of this enzyme either on the outer or the inner leaflet of the vesicle, respectively. To this end, exogenous SMase has been shown to induce in ATP-depleted macrophages and fibroblasts pinching off into the cytoplasm of approximately 400 nm diameter vesicles from the plasma membrane [15]. There is evidence that budding and fusion may happen without assisting proteins such as clathrin [16]. The mechanistic basis of the above morphological transition induced by SMase can be readily rationalized in forms of the distinctly different biophysical characteristics of sphingomyelin and ceramide. In brief, while sphingomyelin is miscible in fluid phosphatidylcholine bilayers, ceramide is laterally segregated [17, 18]. Accordingly, in the course of action of SMase on mixed sphingomyelin/phosphatidylcholine membranes the generated ceramide becomes enriched into microdomains [19]. In terms of physical chemistry this represents an isothermal transition triggered by an enzymatic reaction, causing a shift in the lipid phase diagram from the fluid disordered phase (l_d_) into the two phase region, consisting of l_d_ and solid ordered (l_s_) phases, the latter being enriched of ceramide [20].

The lateral segregation of ceramide is likely to result from an efficient intermolecular hydrogen bonding between the small and weakly hydrated ceramide headgroups [19–23]; their tight lateral packing becomes allowed after the loss of steric repulsion on the headgroup level due to the cleavage by SMase of the strongly hydrated phosphocholine moiety. Tight packing is further augmented by chain-chain van der Waals interactions and attenuated trans→gauche isomerization. Ceramide is readily expected to facilitate the formation of macroscopic invaginations in the membrane [14] by imposing membrane negative spontaneous curvature, the latter ultimately manifesting as the formation of the inverted hexagonal phase H_II_ by this lipid [24, 25]. Because of the interfacial area per molecule, for ceramide is significantly smaller than for sphingomyelin [21], the asymmetric enzymatic formation of ceramide in a bilayer either in the outer or inner leaflet also causes an area difference between the two monolayers. These features, the area difference between the adjacent monolayer leaflets and the negative spontaneous curvature of ceramide containing domains, together with their high bending rigidity provide the driving force for the observed vectorial formation of vesicles in the membrane upon the action of SMase [14]. Product diffusion, domain formation, and vectorial budding into the substrate vesicle interior were seen also when an immobilized SMase was acting on sphingomyelin containing giant liposome membranes [26]. The latter study confirms that it is the molecular properties of the reaction product and not the lipid-protein interaction* per se* that is providing the driving force.

Prompted by the above consequences of the SMase reaction we have suggested that microbes harboring this enzyme on their outer surface and contacting the extracellular leaflet of the plasma membrane of their target cell would induce the above phase change with segregation of ceramide and pinching of the microbe containing “autoendocytotic” vesicles into the cytoplasm of the host cell [14]. Accordingly, it was of interest to search protein sequence databases for possible identification of microbes, which could potentially utilize this mechanism. Because of the characteristic features of the entry of the genus* Chlamydia* into their host cells these microbes were of particular interest. These microorganisms have an intriguing and unique developmental growth cycle. In brief, the latter begins when the infectious, metabolically inactive, and relatively small (0.3 *μ*m in diameter) elementary bodies (EB) attach to the host cell surface, there after somehow stimulating their uptake into the host inside plasma membrane derived vacuoles called inclusions [27, 28]. Several host receptors and surface ligands of EB have been suggested to be involved in their attachment and internalization [29–33]. Yet, the detailed molecular mechanisms of the entry of* Chlamydia* into its host cells have remained elusive. Following their internalization the EB differentiate within a few hours into larger (about one *μ*m in diameter) and metabolically active reticulate bodies (RB). Subsequent replication and the infectious cycle are completed in 2-3 days, when the bacteria have again differentiated to EB, which are released from the host into the extracellular media [27]. The infectious* C. pneumoniae* EB contain the genomic DNA with a coding capacity of approximately 1000 genes and are resistant to environmental stresses, allowing their survival outside of the host cells.

We searched* in silico* the putative surface proteins of* C. pneumoniae* [34] for potential SMase sequences. More specifically, we aligned the sequences of the suggested outer membrane proteins of* C. pneumoniae* with the sequences of a neutral SMase reference group (Table 1). Interestingly, Tr*|*Q9Z8N8*|*Q9Z8N (CPn0300, an omp85 analog) of* C. pneumoniae* scored very well (73/100) in the T-coffee alignment, bearing a high degree of overall structural similarity to the human neutral SMase sp*|*O60906*|*NSMA. The alignment of the latter and Tr*|*Q9Z8N8*|*Q9Z8N revealed strong similarities in the proton binding and substrate recognition areas of SMases. The sequence of Tr*|*Q9Z8N8*|*Q9Z8N further includes a general phosphate binding domain (IMP dehydrogenase/GMP reductase domain), similar to that in alkaline SMases. These studies were complemented by the demonstration of SMase activity in* C. pneumoniae* EB* in vitro*. A pretreatment of the host cell plasma membrane by an exogenously added SMase attenuated the entry and/or growth of* C. pneumoniae* in these cells.

## 2. Materials and Methods

### 2.1. Materials

Bodipy-sphingomyelin (Bdp-SM), Bodipy-ceramide (Bdp-cer), and Amplex Red kit were obtained from Molecular Probes (Eugene, OR). The purity of the above lipids was verified by thin-layer chromatography (TLC) on silicic acid-coated plates (Merck, Darmstadt, Germany) developed with chloroform/methanol/water (65 : 25 : 4, by vol.). The concentrations of Bdp-SM and Bdp-cer in chloroform were determined spectrophotometrically using 77,000 cm^−1^ and 91,000 cm^−1^, respectively, for their molar extinction coefficients. [^35^S]-cysteine/methionine was obtained from Amersham Biosciences. Proanalysis grade solvents were from Merck, SMase from* Staphylococcus aureus* from Sigma, and other chemicals from standard sources.

### 2.2. Sequence Comparisons and Alignments

Sequences of putative outer surface proteins of* C. pneumoniae* [34] were aligned with those in our SMase reference group (Table 1). The latter was randomly selected from all known SMase sequences, with a high degree of overall diversity regarding subfamilies and species. The outer membrane proteins of* C. pneumoniae *that scored best with this reference group were further aligned pairwise with the SMase of greatest resemblance in the reference group. Subsequently, the subfamily of the aligned SMase was aligned against the protein candidate. Special attention was issued to the conserved functionally important sequences. T-coffee (version 2.88) was chosen for this investigation because it has been demonstrated to be more accurate than ClustalW for sequences with less than 30% identity [35]. This issue was of major concern as in spite of possessing the same catalytic activity the sequence similarity of SMases is weak, including their active sites (see Section 4). Pfam (protein domain family database) motifs [36] in CPn0300 were searched by the algorithm available in Expasy (http://myhits.isb-sib.ch/) for the PROSITE database.

### 2.3. Preparation of* C. pneumoniae* EB

HL cells were grown in Dulbecco modified Eagle's medium (DMEM, Sigma) containing 10% fetal calf serum and 20 *μ*g/mL gentamycin, maintained at 37°C under 5% CO_2_.* C. pneumoniae* isolate Kajaani 6 (K6 [37], originally obtained from Prof. Saikku, University of Oulu, Finland) was propagated in HL cells and purified. In brief, infected cells were harvested and ultrasonically disrupted after the cell debris was removed and the bacteria purified by centrifugation in a meglumine diatrizoate gradient [38]. Aliquots of the purified microbe were stored at −70°C in 0.25 M sucrose, 10 mM sodium phosphate, 4 mM potassium phosphate, and 5 mM L-glutamic acid, pH 7.5 (SPG), until used. For host cell attachment assay, the bacteria were metabolically labeled with [^35^S]-cysteine/methionine [39]. The infective titers of the preparations were determined in HL cell cultures and expressed as inclusion forming units per mL (IFU/mL). When indicated the* C. pneumoniae* EB stocks were inactivated with UV-light (2x of 0.120 joules/cm^2^; Crosslinker CL-508, Techne, Cambridge, UK) and did not contain live bacteria when assessed in cell cultures.

### 2.4. Assay for Sphingomyelinase Activity

Two different assays of SMase activity in EB were employed. Accordingly, we first used a fluorescent sphingomyelin analog, Bdp-SM, as a substrate and monitored the appearance of the corresponding fluorescent ceramide by analysis of the reaction by TLC. EB (20–30 *μ*L, 10^9^ IFU/mL) in SPG was added to 50 *μ*M Bdp-SM in 0.5 mM Hepes, 1.8 mM MgCl_2_, 9 mM CaCl_2_, pH 7.4, or, when indicated, in the SPG buffer in a total volume 0.25 mL with the given ion concentrations. After approximately 48 hrs at 37°C with continuous stirring 1.75 mL of chloroform/water/methanol (33 : 25 : 1, by vol.) was added. The lower chloroform phase was collected and dried under nitrogen. The residue was subsequently dissolved in 40 *μ*L of chloroform and a 20 *μ*L aliquot was applied onto silicic acid coated plates. These were developed with 1,2-dichloroethane/methanol/water (90 : 20 : 0.5, by vol.) and the lipid spots visualized by UV illumination. Bdp-SM and Bdp-cer were used as standards.

SMase activity in EB was measured also by the semiquantitative Amplex Red assay (Molecular Probes, Eugene, OR), following the manufacturer's instructions. This indirect method is based on coupled enzymatic reactions. More specifically, the phosphocholine moiety released by SMase from sphingomyelin is first converted into choline by alkaline phosphatase and further oxidized by choline oxidase to betaine and H_2_O_2_. The latter then reacts with 10-acetyl-3,7-dihydroxyphenoxazine (Amplex Red) in a reaction catalyzed by horseradish peroxidase, generating the fluorescent resorufin, which is detected. The reaction mixture [26] contained in the SPG-buffer and in a total volume of 0.2 mL of 5 mM10-acetyl-3,7-dihydroxyphenoxazine, 2.5 mM sphingomyelin, and 2% Triton X-100 (v/v), together with the required cascade of the above enzymes. Reactions were started at 37°C by the addition of* C. pneumoniae* EB (from 0.5 × 10^7^ to 3 × 10^7^ IFUs, as indicated). SMase from* S. aureus* (0.5 mM) and 10 *μ*M H_2_O_2_ were used as controls. Fluorescence intensities were measured with SPECTRAFluor Plus reader (Tecan AG, Hombrechtikon, Switzerland) using excitation and emission wavelengths of 535 and 590 nm, respectively. Based on comparison with* S. aureus* SMase we could estimate the activity of this enzyme in EB in mU per IFU.

### 2.5. Assay for the* C. pneumoniae* Internalization

To monitor the attachment and internalization of* C. pneumoniae,* confluent HL cell monolayers growing on glass coverslips in 24-well plates were incubated for 30 min at 37°C in serum-free DMEM, further supplemented, when indicated, with 20 or 50 mU/mL of* Staphylococcus aureus* SMase. The cells were then extensively washed with DMEM and inoculated immediately with either* C. pneumoniae* K6 or metabolically labelled* C. pneumoniae* K6 at a multiplicity of infection (MOI) of 0.003–0.1. The cells were centrifuged at 2100 rpm at room temperature and then incubated in serum-free DMEM at 37°C for 1 h, followed by washing with serum-free DMEM. To remove attached but not internalized bacteria, the cells were subjected to a mild treatment with trypsin [40] and washed with serum-free DMEM. The cells suspended in PBS were mixed with scintillation cocktail (Optiphase HighSafe3, Wallac, Perkin Elmer life sciences, Turku, Finland) and the radioactivity quantitated by a liquid scintillation counter (1450-Microbeta, Wallac, Turku, Finland). To monitor the growth of internalized* C. pneumoniae*, the inoculated cultures were further incubated for 48 h at 37°C in complete DMEM containing 0.5 *μ*g/mL cycloheximide after the monolayers were fixed with methanol and the chlamydial inclusions were stained with fluorescein-labeled anti-*Chlamydia* antibody (Biorad, Hercules, CA). The inclusions were counted in 20 random 40x fields per coverslip. Data from three parallel wells were combined to calculate the mean and standard deviations.

### 2.6. Cloning of CPn0300 and* C. muridarum* TC_512

Genomic DNA was prepared from* C. pneumoniae* using MagNA Pure Compact nucleic acid isolation kit (Roche Applied Science). The CPn0300 was amplified by PCR and cloned into the BamHI-XhoI site of pGEX-4T-3 vector (GE Healthcare Life Sciences, Uppsala Sweden). Sequencing the cloned plasmid DNA using pGEX-specific primers verified that the insert was CPn0300. The recombinant plasmid was transformed into* E. coli* JM109 and BL-21. The corresponding gene from* C. muridarum* (TC_512) was amplified by PCR and cloned into the SalI-NotI site of the pGEX-4T-3 vector. The expression of CPn0300 and TC_512 was induced with 0.5 mM IPTG. The* E. coli* cells were harvested at 3 hours postinduction by centrifugation (1500 g, 15 minutes) and washed with 10 mM Tris-HCl (pH 8.0) containing 0.1 M NaCl and 1 mM EDTA. Cells were suspended in 25 mM Tris-HCl (pH 8.0) containing 50 mM glucose and 10 mM EDTA. Egg lysozyme was added to the suspension (final concentration 100 *μ*g/mL) and the mixture was incubated for 10 minutes. 10 mM Hepes-NaOH (pH 7.5) containing 0.25 M sucrose, 1 mM EDTA, and protease inhibitor cocktail was added to cell suspension and the resulting mixture was sonicated five times with a probe-type sonicator at 20 W for 10 minutes. The cell lysates were analyzed for SMase activity by using Amplex Red assay. To assess the production of recombinant proteins, the induced cell lysates were analyzed by SDS-PAGE.

## 3. Results

Amplex Red assay was used to confirm SMase activity in* C. pneumoniae* and for a rough quantitation of this enzymatic activity (Figures 4(a), 4(b), and 4(c)). In this method, the phosphocholine headgroup released by SMase is converted in a cascade of coupled enzymatic reactions to a fluorescent end product thus allowing monitoring of the progress of the hydrolytic reaction by the increment in fluorescence with time. Based on the activity of* S. aureus* SMase as an internal standard we could estimate SMase activity to be roughly 0.3 pU per IFU. The measured activity was proportional to the amount of bacteria added (Figure 4(a)). The presence of 9 mM Ca^2+^ enhanced the activity (Figure 4(b)), whereas 2 mM Mg^2+^ was slightly inhibitory (Figure 4(c)). Reduced activity was measured for EB maintained for 5 min at 60°C or 90°C (Figure 7).

We then investigated if prior hydrolysis of the host cell plasma membrane sphingomyelin by exogenously added SMase would affect the infection of the cells by* C. pneumoniae*. Interestingly, preincubation of HL cells with* S. aureus* SMase prior to their inoculation with EB attenuated the bacterial entry and/or growth. This effect was proportional to the amount of SMase added (Figure 5(a)). As this attenuation could also result from an apoptotic challenge of the cells due to SMase we checked if the pretreatment of cells with SMase caused activation of caspase-3. However, no increase in the activity of this enzyme was evident (data not shown). This result proves that the apoptotic challenge of formed ceramide cannot be the reason for attenuation. Interestingly, we could not see the attenuation if HL cells were infected with mouse Chlamydia,* Chlamydia muridarum* (data not shown). The above was confirmed in experiments where radioactive EB were used to quantify the internalization by cultured cells, with heparin preventing the binding of EB to the host cell ([41], Figure 5(b)). Our data thus suggest that the availability of sphingomyelin on the external surface of host cells could represent a limiting factor for* C. pneumoniae* internalization.

**Figure 1:**
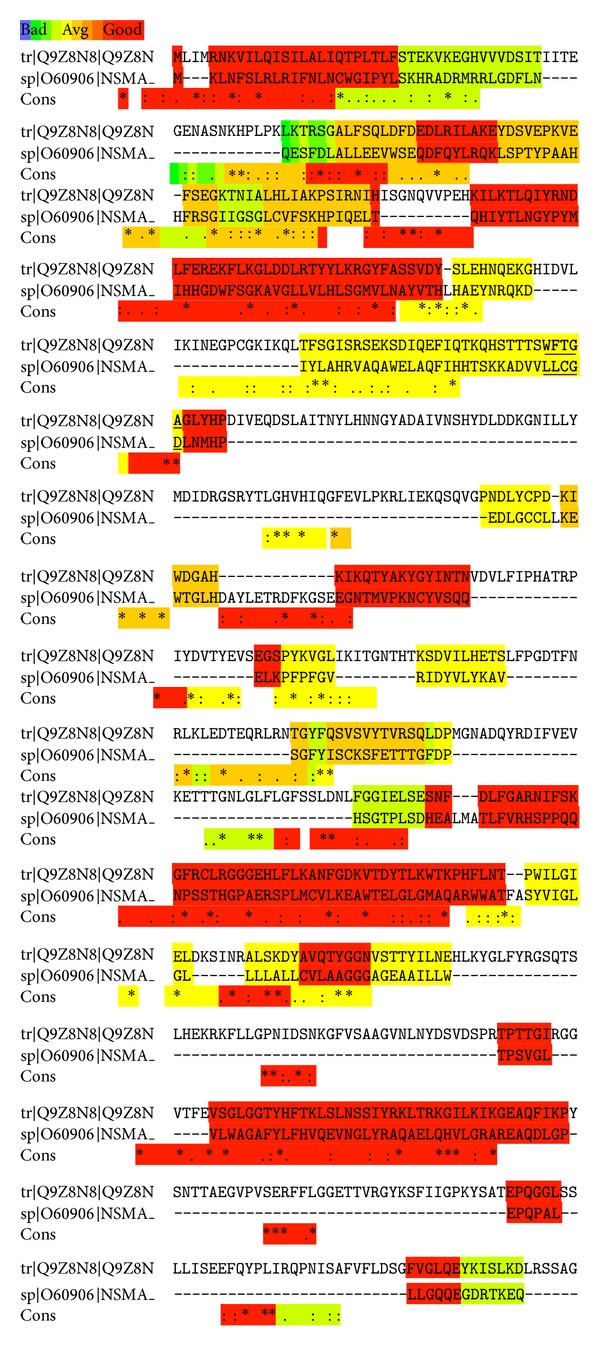
T-coffee alignment of the sequences of tr*|*Q9Z8N8*|*Q9Z8N of* Chlamydia pneumoniae* (CPn0300) and human neutral sphingomyelinase, NSMase sp*|*O60906*|*NSMA. Putative proton binding site and proton acceptor site are underlined (residues 220 to 229 and 463 to 465 in CPn0300, resp.).

**Figure 2:**
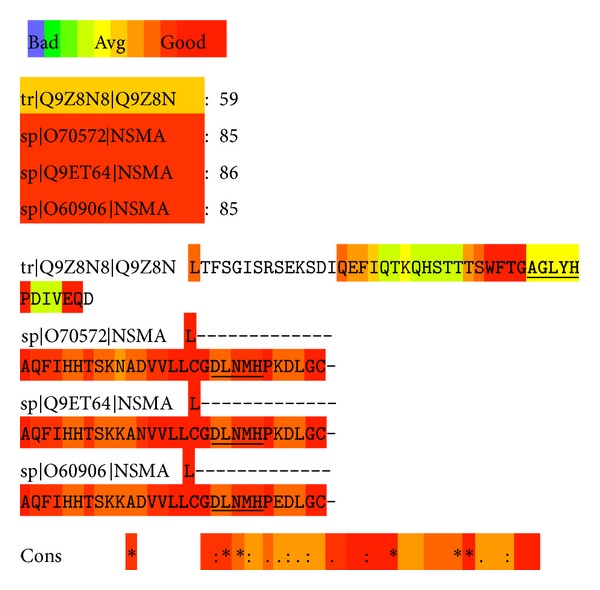
T-coffee alignment of CPn0300 (top) with the sequences involved in proton binding and substrate recognition by type 1 neutral SMases sp*|*O70572*|*NSMA (*Mus musculus*), sp*|*Q9ET64*|*NSMA (*Rattus norvegicus*), and sp*|*O60906*|*NSMA (*Homo sapiens*) as indicated.

Putative* C. pneumoniae* outer surface proteins [34] were aligned by T-coffee with a reference group of sphingomyelinases from diverse species and subfamilies ranging from bacterial enzymes to human neutral sphingomyelinases (Table 1). Only the entire sequences of these proteins were included. T-coffee scores sequence alignments in a range of 1–100, with the range 70 to 100 regarded as good while values below 50 are poor, revealing a lack of similarity. Interestingly, the* C. pneumoniae* protein Tr*|*Q9Z8N8*|*Q9Z8N (CPn0300, an omp85 analog) scored 73. In the alignment of the active site containing domains of the three neutral SMases highest sequence similarity of CPn0300 was evident with the human neutral SMase sp*|*O60906*|*NSMA (Figure 1). Of particular interest are the similarities in the sequences known to be involved in proton binding [42] and substrate recognition [43] by the NSMase protein family, the highly conserved histidine-proline loop [42] being found in the latter domain (Figure 2). Against the selected NSMase protein subfamily CPn0300 scored 40, which was roughly the same as the similarity of the NSMase of baker's yeast (sp*|*P40015*|*ISC1_YEAST) to the selected NSMase protein subfamily. Two human enzymes, NSMase1 and NSMase2, have been identified, with the former representing a distant homolog of bacterial SMases [44]. Comparison of these two SMases with CPn0300 of* C. pneumoniae* suggests the latter to belong to the group 1 enzymes (Figure 1). Interestingly, Pfam is a large collection of multiple sequence alignments and hidden Markov models covering many common protein domains and families. The Pfam motif of IMP dehydrogenase/GMP reductase (http://myhits.isb-sib.ch/cgi-bin/motif_scan) in CPn0300 was found beginning of amino acid 67 to 388 [36].

**Figure 3:**
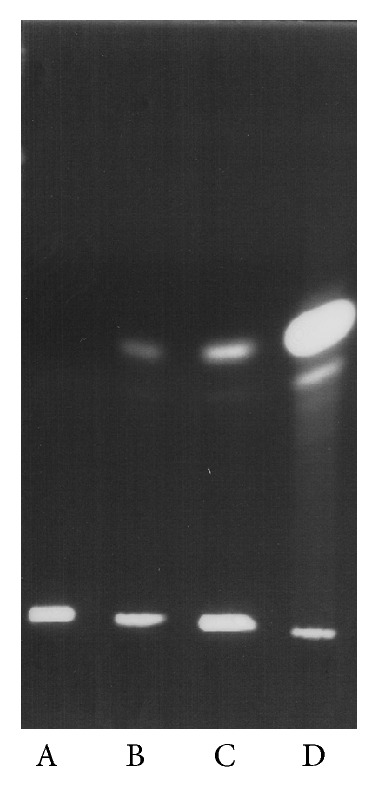
TLC analysis on silicic acid coated plates of the hydrolysis of the fluorescent Bdp-sphingomyelin (*lane *A) to Bdp-ceramide (*lane *D). Formation of fluorescent ceramide from Bdp-SM (50 *μ*M final concentration) in 0.5 mM Hepes, 1.8 mM MgCl_2_, and 9 mM CaCl_2_; pH 7.4 was monitored after the addition of 3.3 × 10^7^ (inclusion forming units (IFUs) (*lane* B) and 6.6 × 10^7^ IFUs of intact* C. pneumoniae *(*lane *C) in the same buffer. Incubation for 48 hrs at 37°C was used to ensure sufficient product formation so as to allow the detection of ceramide upon TLC despite losing some of this lipid in extraction. The TLC plates were developed with 1,2-dichloroethane/methanol/water (90 : 20 : 0.5, by vol) and the lipids localized by illumination with a UV lamp.

**Figure 4:**
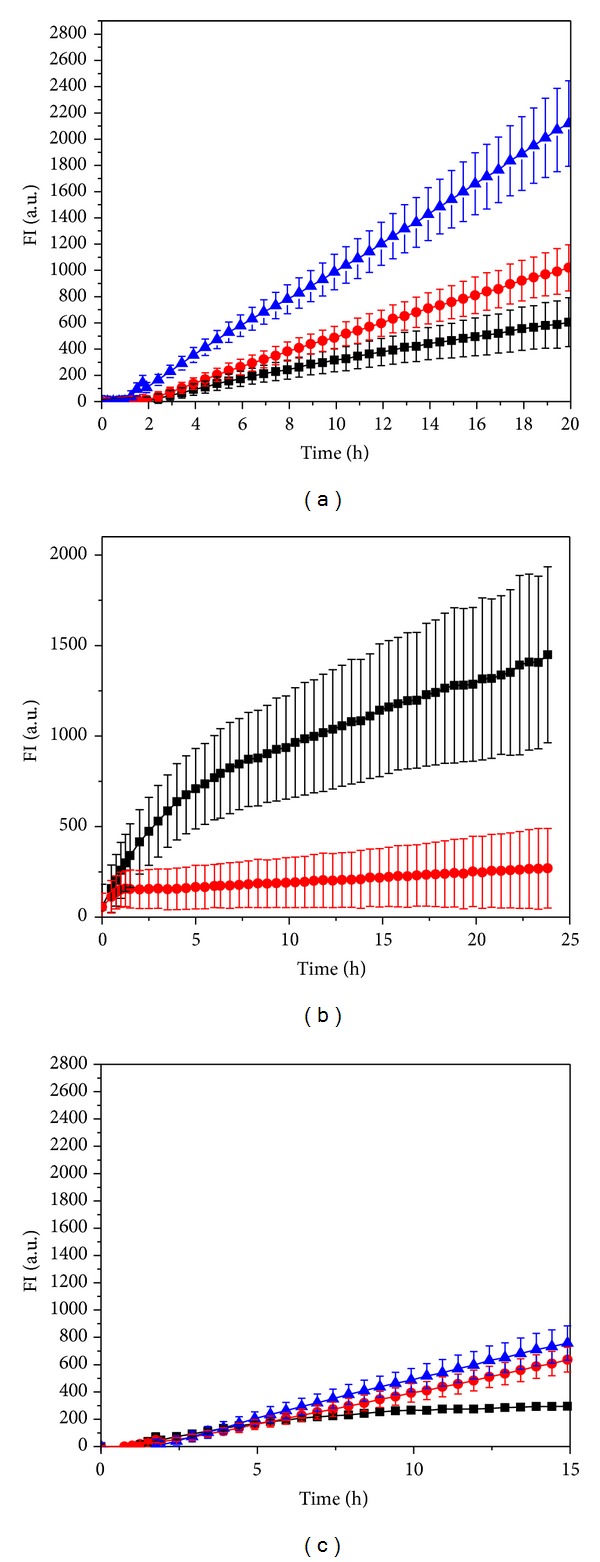
(a) Time courses for the increase in resorufin fluorescence FI a.u. (fluorescence intensity, arbitrary units) due to* C. pneumoniae* is measured by the Amplex Red assay. The reactions were started by the addition of 0.5 × 10^7^ (■), 1 × 10^7^ (●), and 3 × 10^7^ (▲) inclusion forming units (IFUs) of bacteria in SPG and in a total volume of 200 *μ*L at 37°C. (b) SMase activity of* C. pneumoniae* measured by the Amplex Red assay. The reactions were started by the addition of 3 × 10^7^ IFUs (in 30 *μ*L of SPG buffer) in the presence of either 9 mM CaCl_2_ (■) or 9 mM CaCl_2_ and 35 mM EDTA (●) and in a total volume of 200 *μ*L. Temperature was 37°C. (c) SMase activity of* C. pneumoniae* in SPG buffer (▲) and with either 2 mM MgCl_2_ (■) or 2 mM MgCl_2_ and 4 mM EDTA (●). The Amplex Red assays for SMase were started by the addition of 1.5 × 10^7^ IFUs of bacteria, incubated in a total volume of 200 *μ*L at 37°C.

**Figure 5:**
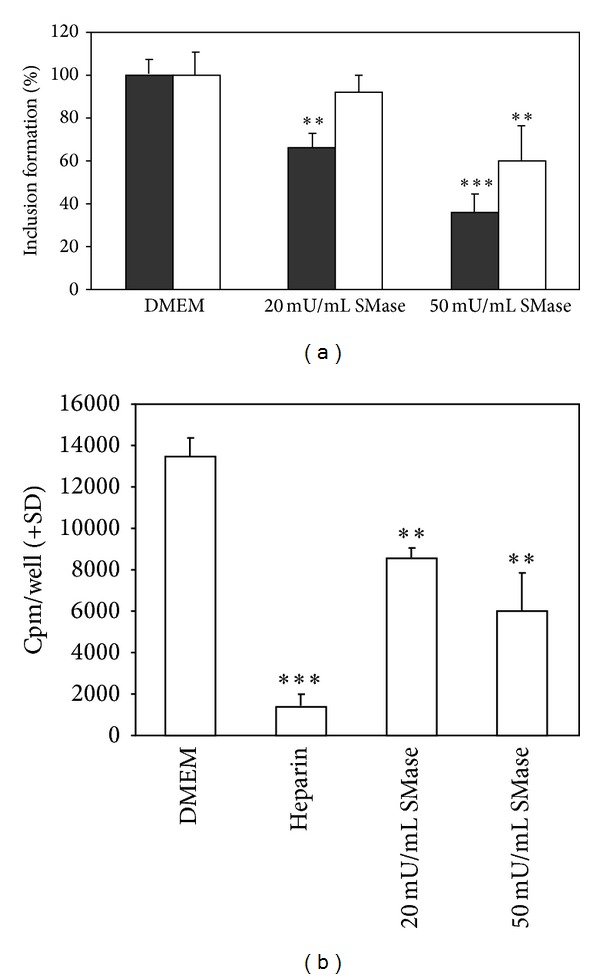
Impact of a pretreatment of the host cells by exogenous SMase on the entry and growth of* C. pneumoniae.* (a) HL cells were incubated for 30 min at 37°C with either 20 or 50 mU/mL of* S. aureus* SMase, as indicated, either before (black bars) or after (white bars) their inoculation with* C. pneumoniae* (0.1 × 10^6^ IFUs). Growth of* C. pneumoniae* was detected at 48 hours postinoculation. (b) Internalization of* C. pneumoniae* by HL cells pretreated with SMase (20 or 50 mU/mL, final concentration), followed by inoculation with metabolically labelled bacteria. Radioactivity in cells was measured after inoculation and trypsin treatment. Heparin (500 *μ*g/mL) inhibits attachment of bacteria to the host cells and was employed as a negative control. The error bars indicate standard deviations from triplicate wells. Student's* t*-test gave values of **P < 0.01 and ***P < 0.001 for the difference between the cells treated with SMase and controls in DMEM.

Following the tentative identification of CPn0300 as a potential SMase it was of interest to study if isolated* C. pneumoniae* EB possess this enzymatic activity. This was first assessed by incubating purified EB with sphingomyelin bearing the covalently linked Bodipy fluorophore in the N-acyl chain. After 48 hrs of incubation at 37°C the lipids were isolated on TLC and visualized by UV-illumination (Figure 3). Formation of ceramide was evident in the presence of* C. pneumoniae* EB and its content did depend on the amount of bacteria added, as shown by visual inspection of the lanes for the amount of ceramide formed in the presence of approximately 33 × 10^6^ and 66 × 10^6^ IFUs (Figure 3,* lanes B* and* C*, resp.).

The importance of host cell surface sphingomyelin to the internalization process of* C. pneumoniae* was studied. Pretreatment of host cells with exogenously added SMase decreased the formation of chlamydial inclusions in the* C. pneumoniae* infection. This phenomenon correlated with the amount of SMase added. With 50 mU/mL SMase, the formation of inclusions was inhibited by more than 50%. Pretreatment of the host cells by SMase consumed the SM available on the plasma membrane of HL cells thus decreasing chlamydial intake. Earlier, Allan and Quinn [45] showed that after SMase hydrolysis on the surface of BHK cells, it took 3 hours to get SM content back to normal on the plasma membrane. They also showed that the SM of an inaccessible pool (inside the cell) will not equilibrate with the plasma membrane pool, even after prolonged incubation. Instead, Triton X-100 treatment of the cells caused an almost complete breakdown of total BHK sphingomyelin.

Because sphingomyelinase activity in* C. pneumoniae* has not been previously reported, the membrane proteins potentially responsible for the SMase activity were further investigated.

**Figure 6:**
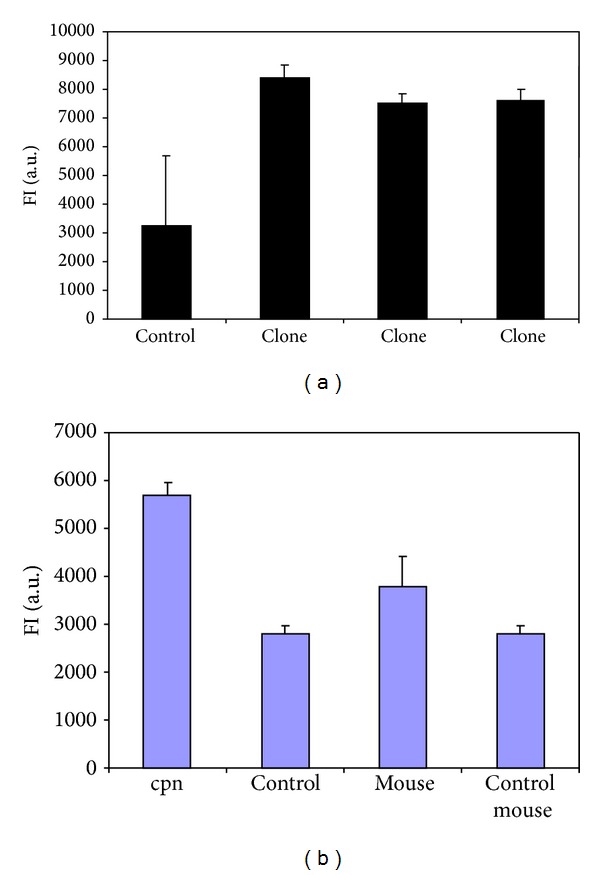
Measurement of SMase activity from lysates of JM109 transformed with recombinant pGEX-4T-3 by Amplex Red assay. Resorufin fluorescence FI a.u. (fluorescence intensity, arbitrary units) was measured.* E. coli* JM109 transformed with recombinant pGEX-4T-3 were induced with 0.5 mM IPTG. Three hours later, the cells were lysed as described in Section 2. Ten 10 *μ*L of the lysate was used in the measurement done after 30 minutes of incubation at 37°C. (a) SMase activity of free different clones of CPn0300 was measured. (b) SMase activity of* E. coli* lysates expressing CPn0300 and* C. muridarum* TC_512 was measured. Controls express plain vector.

To construct a recombinant plasmid for expression of a* C. pneumoniae* sphingomyelinase coding sequence*, C. pneumoniae* DNA was amplified using CPn0300 specific primers. After PCR, the size of the PCR product was verified by agarose gel electrophoresis. A product of 2387 bp was detected, as expected. The PCR product was digested with BamHI and XhoI and ligated to a pGEX-4T-3 vector and the recombinant plasmid was transformed into* E. coli*. Plasmid DNA was purified from the transformed* E. coli* and digested with BamHI, XhoI, and PstI. The restriction enzyme analysis suggested that the insert was CPn0300. This was further confirmed by sequencing the plasmid DNA using pGEX-specific primers. Finally, induced cell lysates expressing CPn0300 that ran on the SDS-page showed production of a glutathione-s-transferase (GST)-CPn0300 fusion protein of approximately 110–115 kDs in size.* E. coli* transformed with parental pGEX-4T-3 was used as a control for GST expression (Figures 6(a) and 6(b)).

Sphingomyelinase activity present in the cell lysates was measured using Amplex Red assay. The activity was significantly higher in the lysates of* E. coli* expressing CPn0300 than in cells having the plain vector or in lysates expressing* C. muridarum* TC_512 (Figures 6(a) and 6(b)). This data is in consistence with the fact that we could not see inhibitory effect of SMase pretreatment on the formation of inclusion bodies in HL cells when HL cells were infected with* C. muridarum*.

## 4. Discussion

The genus* Chlamydia* includes several speciesof small intracellularly growing bacteria, which can enter and multiply in a variety of human cells [46], including both professional and nonprofessional phagocytes, and cause a variety of diseases in both human and animal hosts [47].* C. trachomatis* causes sexually transmitted infections and blinding trachoma, while* C. psittaci* is a pathogen primarily for birds and mammals, being only occasionally transmitted to humans.* C. pneumoniae* is a common human pathogen causing respiratory tract infections associated with chronic conditions such as atherosclerosis and coronary heart disease [48, 49], asthma [50], chronic bronchitis [51], reactive arthritis [52], and Alzheimer's disease [53].

**Figure 7:**
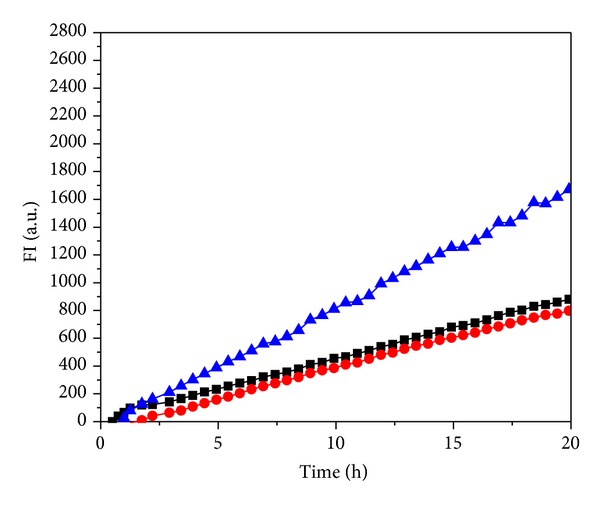
Effect of heat treatment on the SMase activity of* C. pneumoniae*. Bacteria were incubated for 5 min at either 60 (■) or 90°C (●), and bacteria maintained at 37°C were used as a control (▲). Amplex Red assays were started by adding 1 × 10^7^ IFUs of bacteria and incubated in a total volume of 200 *μ*L at 37°C. Resorufin fluorescence FI a.u. (fluorescence intensity, arbitrary units) was measured.

The viability of* Chlamydia* and its surface structure are important to the attachment and entry of this microbe into its host cells. The binding of* C. trachomatis* to host cells increases with temperature between 0 and 37°C [54]. While UV-inactivated* Chlamydia* EB remain capable of entry into mouse fibroblasts [55], heat treatment (i.e., 5 min at 60 or 90°C) of EB reduces their uptake [54]. Our current study demonstrates SMase activity in UV inactivated EB (data not shown) and decreased activity in heat treated EB (Figure 7). As far as we are aware this is the first report on an enzymatic activity in chlamydial EB. The substrate for SMase, sphingomyelin, is an abundant phospholipid in eukaryote membranes and it is enriched in the outer leaflet of the plasma membrane [56] thus being readily accessible to invading microbes. The observed transient externalization of phosphatidylserine accompanying the entry of* C. pneumoniae* into epithelial, endothelial, granulocytic, and monocytic cells [57] could reflect an accumulation of ceramide in the host cell membranes upon their contact with EB. Interestingly, the exposure of PS on the outer cell surface of host cells has been reported upon infection by* P. falciparum* [58] and SMase activity of this parasite has been concluded to be essential for the infection of erythrocytes [7]. In addition to its role in apoptosis [59], ceramide has been shown to be intimately involved also in the signaling cascades underlying inflammation [1, 2, 4, 5]. Precursor of ceramide, sphingomyelin, has a putative role in inclusion body formation and intracellular trafficking [60]. It seems feasible that by increasing the cellular levels of ceramide in the affected host tissues the presence of* C. pneumoniae* could perturb the metabolic state of cells and tissues and thus contribute to the pathophysiology of the secondary disorders connected to* Chlamydia* infection.

In general, the sequence similarity between different SMases such as those in our reference group (Table 1) is weak, in spite of the fact that these proteins possess the same catalytic activity. Because of this high variability of the SMase sequences their identity goes easily unnoticed when stringent similarity searches are used. Nevertheless, the assessment of CPn0300 with Nomad (Neighborhood Optimization for Multiple Alignment Discovery, see Expasy, http://ca.expasy.org/) reveals an equal degree of sequence similarity to the endonuclease/exonuclease/phosphatase domain of SMases as between most nonmammalian SMases. Total alignment with N-SMase gave best overall results. Interestingly, also the pfam_ls:IMPDH (IMP dehydrogenase/GMP reductase domain) motif is present in CPn0300, between residues 67 and 388. This domain belongs to the common phosphate binding site in the TIM barrel superfamily and includes various phosphatases and lipases [61]. CPn0300 thus appears to possess the same active domain site as alkaline sphingomyelin phosphodiesterases. The catalytic domain of human neutral SMases belongs to the endonuclease/exonuclease/phosphatase family (http://www.uniprot.org/uniprot/O60906). Our sequence analysis of* C. pneumoniae* suggests the omp85 homolog CPn0300 to be a possible candidate responsible for the SMase activity measured for this microbe. Along these lines it seems possible that SMase activity could be found in other bacterial outer membrane proteins, the omp85 family in particular. However, it is as well possible that some of the SMases are not intrinsic SMase of* C. pneumonia *but carried on the top of the capsid from the host cell. Whichever the case, our data also provide support to the view that this enzymatic activity is involved in the host cell membrane manipulation of* C. pneumoniae* that is important in the host cell internalisation and externalisation. In keeping with this notion, omp85 specific antibodies have been shown to inhibit chlamydial infectivity* in vitro* [62]. While the function of CPn0300 [34] has remained elusive, this putative outer surface protein is highly conserved between the different* C. pneumoniae* strains [63]. The proteins in the omp85 family have been shown to have various membrane affecting roles. In* E. coli* YaeT (an ortholog of omp85) has a critical role in the outer membrane assembly [64]. PorB, a component of the chlamydial outer membrane, has pore forming activity [65]; however, this activity was not detected for chlamydial omp85 when incorporated into liposomes [62].

Enzymatic manipulation of membrane lipids can be used for a number of different purposes [22]. Along these lines the most common human pathogen* S. aureus* secretes SMase [66], which is lytic to the host cells thus allowing the microbe to use the host tissues for nutrition. In contrast, the life cycle of* Chlamydia* requires the host cell to remain viable, so as to allow the EB→RB conversion and subsequent replication within the host. Accordingly, only a relative low SMase activity is adequate to avoid triggering of apoptosis in the host too early, before completion of the replication of the invading* Chlamydia*. Accordingly, it seems feasible that EB-catalyzed formation of ceramide could trigger the death of the host after the completion of the replication cycle. Finally, it is possible that different mechanisms of entry are utilized by different* Chlamydiae* as well as in the entry into different cell types. Understanding these processes would shed light on pathogenesis of chlamydial infections and potentially facilitate the development of novel means for therapeutic intervention.

## Figures and Tables

**Table 1:** SMase reference group.

sp|P69502|SMAL_LOXGA	SMase loxnecrogin	EC 3.1.4.41	*Loxosceles gaucho* (spider)
sp|Q7Z1Y7|SMAD_LOXAR	SMase D precursor	EC 3.1.4.41	*Loxosceles arizonica* (arizona brown spider)
sp|P83045|SMA1_LOXIN	SMase P1 precursor	EC 3.1.4.41	*Loxosceles intermedia* (spider)
sp|P17627|PHL_LEPIN	SMase C precursor	EC 3.1.4.12	*Leptospira interrogans *
sp|P80924|PHLC_STAIN	SMase C	EC 3.1.4.12	*Staphylococcus intermedius *
sp|P09978|PHLC_STAAU	Phospholipase C precursor	EC 3.1.4.3	*Staphylococcus aureus *
sp|P09599|PHL1_BACCE	SMase C precursor	EC 3.1.4.12	*Bacillus cereus *
sp|O60906|NSMA_HUMAN	NSMase 1	EC 3.1.4.12	*Homo sapiens *
